# Wu-tou decoction attenuates neuropathic pain via suppressing spinal astrocytic IL-1R1/TRAF6/JNK signaling

**DOI:** 10.18632/oncotarget.21638

**Published:** 2017-10-06

**Authors:** Chao Wang, Xiangying Kong, Chunyan Zhu, Chunfang Liu, Danni Sun, Qionghong Xu, Zhiyun Mao, Qingxia Qin, Hongchang Su, Danqiao Wang, Xiaoliang Zhao, Na Lin

**Affiliations:** ^1^ Institute of Chinese Materia Medica, China Academy of Chinese Medical Sciences, Beijing 100700, China; ^2^ Experimental Research Center, China Academy of Chinese Medical Sciences, Beijing 100700, China

**Keywords:** Wu-tou decoction (WTD), neuropathic pain (NP), glia-mediated neuroinflammation, astrocytic IL-1R1/TRAF6/JNK signaling, anti-hyperalgesia

## Abstract

Neuropathic pain (NP) caused by nerve injuries continues to be an intractable challenge due to inadequate therapeutic strategies. Recent study demonstrated glia-induced neuro-inflammation in the spinal cord, especially the activation of astrocytes, plays an essential role in the development of NP, which opens new avenues for NP treatment. In this study, we explored the anti-hyperalgesia properties of Wu-tou decoction (WTD) and showed that WTD potently attenuates mechanical allodynia and heat hyperalgesia in lumbar 5 (L5) spinal nerve ligation (SNL)-induced NP without noticeable side effect or affecting basal pain perception of mice. Mechanistically, initial targets screening tests indicated WTD's analgesic action may be centrally mediated within the spinal cord, which further verified by its inhibitory actions on glia-releasing factors of IL-1β, CCL2 and CXCL1. Meanwhile, WTD significantly reduced spinal IL-1R1, TRAF6 expressions, p-JNK levels, and number of GFAP/IL-1R1, GFAP/TRAF6, GFAP/p-JNK positive astrocytes in the superficial lamina of spinal cord. Additionally, co-administration of IL-1Ra increased the anti-hyperalgesia effects of WTD and further decreased CCL2 and CXCL1 expressions, while no synergistic effects were detected when TRAF6 or JNK inhibitors were co-administrated with WTD. Thus, our data suggested that the effective inhibition of spinal astrocytic IL-1R1/TRAF6/JNK signaling (especially IL-1R1) contributes, at least in part, to WTD's anti-hyperalgesia action. It also indicates that WTD might be a promising candidate for the treatments of chronic pain, especially under NP-related neurological disorders.

## INTRODUCTION

Neuropathic pain (NP) resulting from nerve damage is a devastating disease which mainly characterized by mechanical allodynia and thermal hyperalgesia [1–3]. This excruciating conditions create considerable suffering for people affected, and bring extreme economic burden for the individual and for the community [4, 5]. It is generally believed that NP is caused by neural plasticity [6–9], which leads to neuronal-targeted drugs such as opioid analgesics, antidepressants, anticonvulsants drugs and calcium channel blockers. However, these analgesics that predominately modulate pain transduction and transmission in neurons have limited effects, and the central nervous system (CNS)-related side effects such as nausea, sedation, drowsiness, and dizziness, as well as development of analgesic tolerance, have greatly limited their clinical applications [10, 11]. NP still remains a major therapeutic challenge and a significant public health issue, and its mechanisms are incompletely understood.

Accumulating evidence suggests that local inflammation (neuro-inflammation) in the spinal cord, which is characterized by temporal activation of microglia (within days), astrocytes (within days to weeks) and production of inflammatory mediators, plays an important role in the initiation and maintenance of NP, especially the activation of astrocytes, a typical pathological characteristic of NP, is crucial for the persistence of pain syndrome [2, 12–16]. Under nerve injury conditions, microglia produces multiple inflammatory mediators such as IL-1β and TNF-α, which mediate signaling from receptor super families, such as tumor necrosis factor receptor (TNFR) and interleukin-1 receptor (IL-1R) by increasing TNF receptor associated factor 6 (TRAF6) expression [17–19], subsequently activate c-Jun N-terminal Kinase (JNK) and induce the production of chemokine such as C–C motif chemokine ligand 2 (CCL2), C-X-C motif chemokine ligand 1 (CXCL1) in astrocytes that are implicated in pain sensitization [20, 21]. Thus, targeting glia-induced excessive neuro-inflammation offers new therapeutic opportunities for NP-related neurological disorders [2, 12–14].

Because of its fewer side effects and abundance of available source, natural products have now been emerging as important therapeutic resources for the development of new analgesics and the management of certain chronic pain states [22]. Wu-tou decoction (WTD), a well-known Traditional Chinese Medicine (TCM) prescription formulated by Chinese medical sage Zhongjing Zhang, consisting of *Radix Aconiti, Herba Ephedrae, Radix Astragali, Raidix Paeoniae Alba and Radix Glycytthizae*, has been therapeutically used to treat inflammatory diseases and joint diseases effectively for more than one millennium in China. Previous studies have demonstrated that WTD possesses anti-inflammatory properties by inhibiting inflammatory cytokines and regulating TLR2/TRAF6/Faslg signaling [23, 24]. Recently, we have found the regulatory effects of WTD in the imbalance of nervous, endocrine and immune systems and its activation effects on PPAR-gamma pathway in complete Freund's adjuvant (CFA)-induced arthritis [25, 26]. Moreover, our recent studies have also indicated that WTD could alleviate chronic inflammatory pain in a transient receptor potential ion channel- (TRP-) related manner [27]. However, little is known about whether WTD produces anti-hyperalgesia or anti-allodynia effects to attenuate NP, and its anti-hyperalgesia characteristics or the underlying mechanisms remain to be elucidated.

Given that astrocyte and astrocyte-expressing molecules in the spinal cord play essential roles in neuro-inflammation and the development of NP [2, 12–16], and WTD has potent anti-inflammatory properties [23–27], we hypothesized that WTD may have alleviating effects on NP via regulation of astrocytic-associating molecules in CNS. Our findings demonstrated that WTD significantly attenuates spinal nerve ligation (SNL)-induced mechanical allodynia and heat hyperalgesia of mice possibly through inhibition of astrocytic IL-1R1/TRAF6/JNK signaling, and the effective inhibition of IL-1R1 maybe the key link that contributes to WTD analgesic action.

## RESULTS

### WTD reversed mechanical allodynia and heat hypersensitivity in SNL-induced mice without noticeable side effects

**Figure 1:**
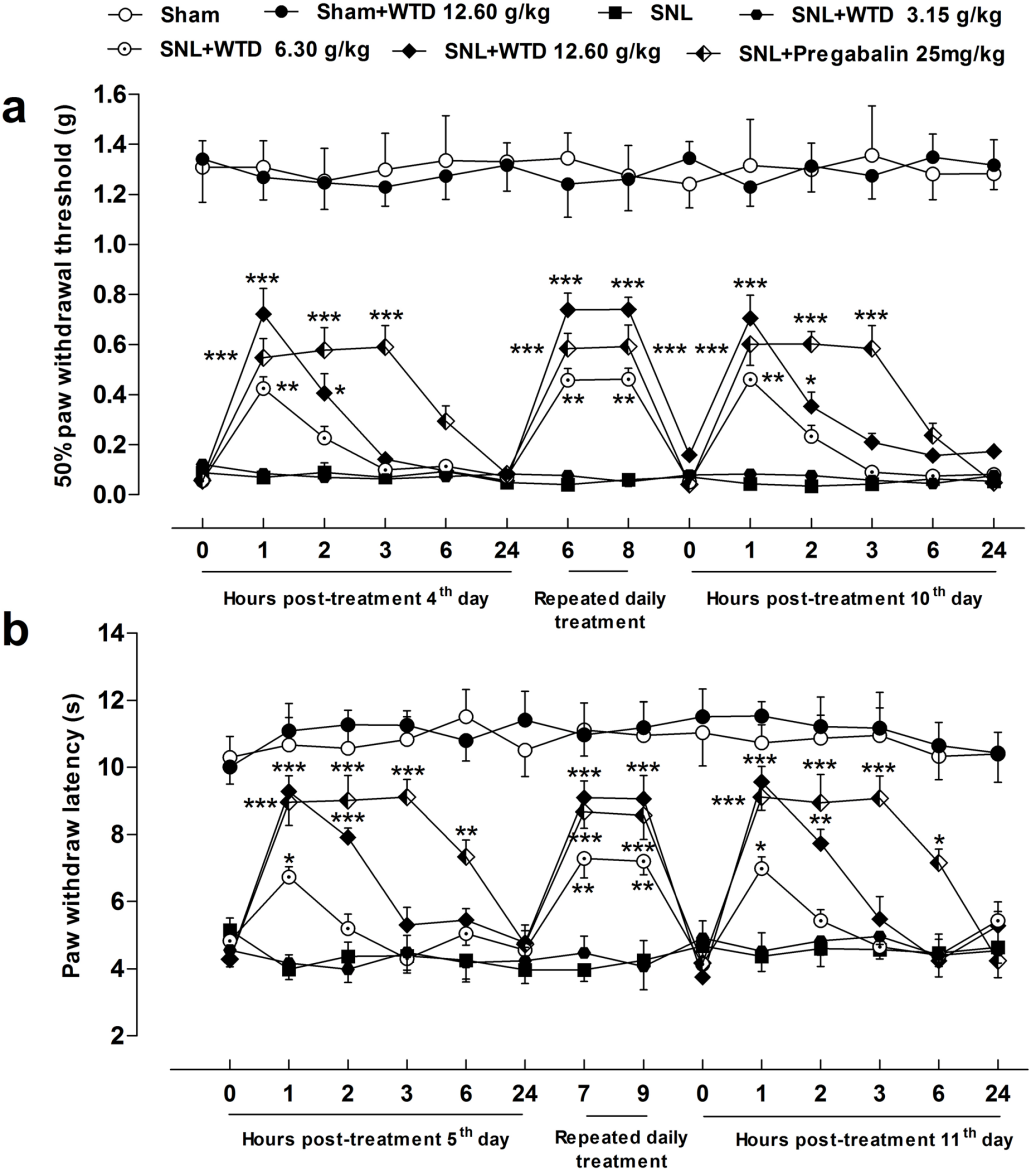
Characteristics of anti-hyperalgesia of WTD on SNL-induced neuropathic pain SNL induced obvious mechanical allodynia and heat hyperalgesia of mice. WTD (6.30-12.60 g/kg) significantly reversed SNL-induced mechanical allodynia **(a)** and heat hyperalgesia **(b)**, the anti-hyperalgesia effects of pregabalin (25 mg/kg, p.o.) were similar to that of high dose of WTD (12.60 g/kg) (a and b). Whereas WTD did not alter the baseline threshold in sham-operated group (a and b). Data are represented as mean ± SEM. (n=6). ^*^P<0.05, ^**^P<0.01 and ^***^P<0.001 *vs*. SNL group, respectively.

Previously, we showed that WTD significantly reversed mechanical and thermal hypersensitivities under chronic inflammatory pain conditions [27], to further investigate the anti-hyperalgesia effects of WTD on NP disorders, a SNL-induced pain model was used. As shown in Figure 1a, 3 days after surgical operation, SNL induced significant mechanical allodynia characterized by the reduced 50% paw withdrawal threshold (PWT) compared to sham group. WTD (6.30-12.60 g/kg) significantly attenuated mechanical allodynia, which lasted for at least 2 hours with optimal inhibitory effect 1 hour post WTD administration, but low dose of WTD (3.15 g/kg) has no obvious anti-allodynia effect. And this effect was maintained for at least 7 days while dug was daily administrated. Importantly, on day 7, WTD (6.30-12.60 g/kg) reduced mechanical allodynia with a time-course effect profile similar to that of the first day, indicating no drug tolerance [28, 29]. Although WTD (6.30-12.60 g/kg) significantly attenuated mechanical allodynia in SNL-induced hypersensitivity, it did not alter the baseline thresholds in sham control group, suggesting WTD has a specific role for NP conditions. Pregabalin (25 mg/kg, p.o.) manifested anti-allodynia effect similarly to that of high dose of WTD (12.60 g/kg, p.o.), but produced longer inhibitory action than WTD (12.60 g/kg, p.o.) when single dose drug administrated.

We further checked the anti-hyperalgesia effects of WTD in a radiant heat plantar test. The behavioral results showed a remarkable inhibition of heat hyperalgesia by WTD (6.30-12.60 g/kg) administration, with similar time-course effect to that of WTD on mechanical allodynia (Figure 1b). Also, WTD (12.60 g/kg) produced no analgesic effect in sham control mice in the radiant heat test, further ruling out the possibilities of drug tolerance [28, 29] and identifying its unique role for NP normalization. Pregabalin (25 mg/kg, p.o.) also significantly alleviated heat hyperanalgesia in the similar manner (Figure 1b).

Since myorelaxant drugs or sedatives can promote changes in motor function, coordination, or induce sedation resulting in false positive results for behavioral analysis in rodents [30], we assessed whether pretreatment of WTD produce such side effects by using a Rota-rod apparatus. As shown in Supplementary Figure 1a, 0, 1, 3, 6 hours post treatments, mice in WTD (3.15-12.60 g/kg) and pregabalin (25 mg/kg) groups did not manifest noticeable impaired locomotor function, while pregabalin (50 mg/kg) significantly reduced the latency time of falling 1 or 3 hours post treatment, indicating WTD (3.15-12.60 g/kg) administration did not produce obvious effect on motor functions. In addition, chronic administration of WTD (3.15-12.60 g/kg) did not induce obvious body weight loss of animals (Supplementary Figure 1b).

### WTD ameliorated formalin- but not hot plate-induced nociception

**Figure 2:**
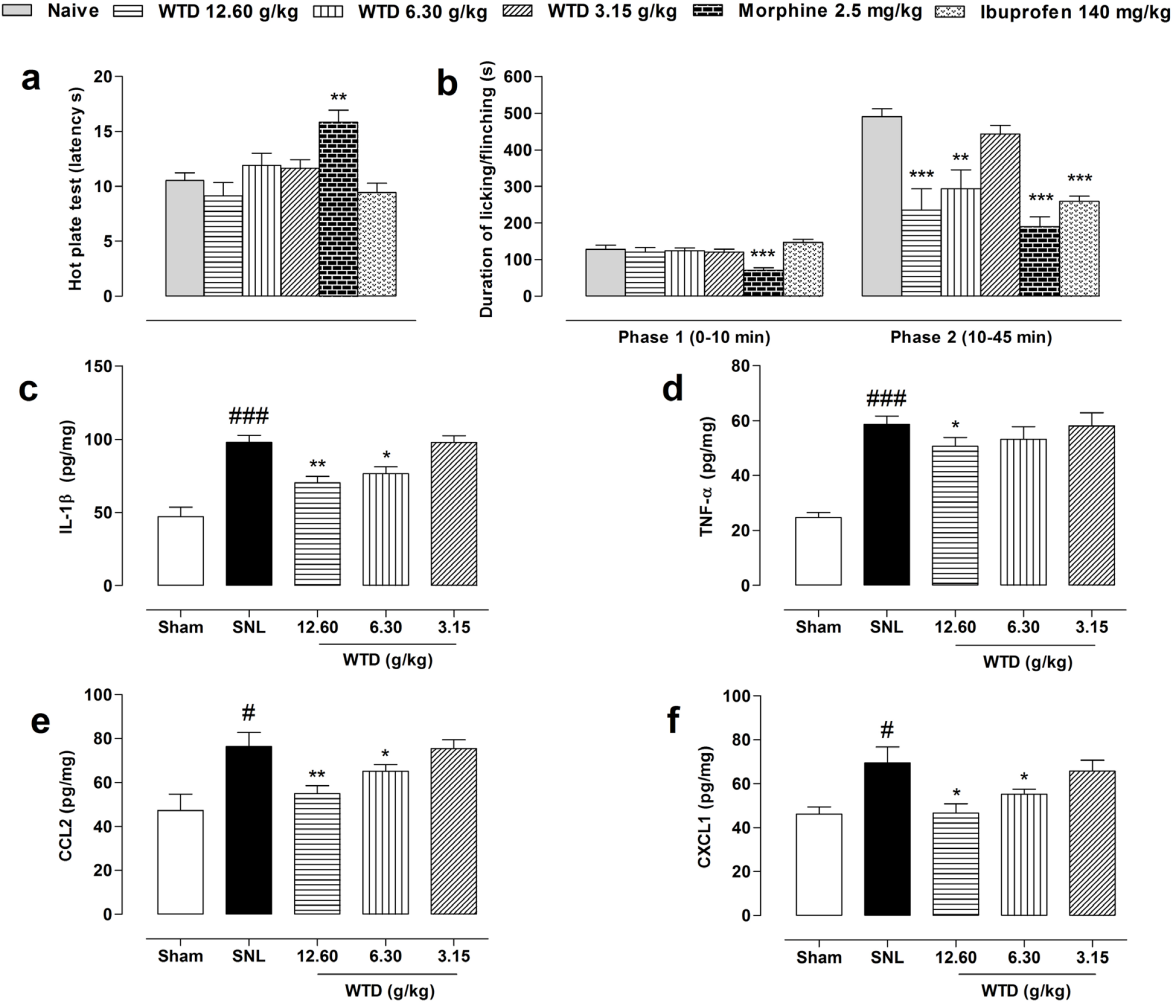
Initial targets screening of WTD analgesic action Oral administration of WTD (3.15-12.60 g/kg) did not increase latency time of nociception in a hot-plate test **(a)**. WTD remarkably reduced the second phase of formalin-induced nociception of naïve mice **(b)**. WTD could also significantly reduced IL-1β **(c)**, CCL2 **(e)** and CXCL1 **(f)**, but with limited inhibitory effect on TNF-α **(d)** in the dorsal horn of L5 spinal cord of SNL mice. Data are represented as mean ± SEM. (n=5-6). ^*^P<0.05, ^**^P<0.01 and ^***^P<0.001 in **(a-b)** *vs*. naïve group; in **(c-f)** *vs*. SNL group; ^#^*P*<0.05, ^###^*P*<0.001 *vs*. sham group.

Next, we asked the mechanism responsible for WTD's anti-hyperanalgesia action of effects. For this, we first screened the possible analgesic targets of WTD action in a hot plat test. Given orally, preemptive administration of WTD (3.15-12.60 g/kg) or ibuprofen (140 mg/kg) did not produce any analgesic effects of mice, while morphine (2.5 mg/kg, s.c.) significantly increased the latency time of nociception in the hot-plate test (Figure 2a), indicating no supraspinal mechanisms of action for WTD [29].

We further checked the analgesic actions of WTD in an acute inflammatory model by subcutaneous application of formalin in ICR mice. Pretreatment of WTD (6.30-12.60 g/kg) significantly decreased Phase II (10-45 minutes) but not Phase I (0-10 minutes) pain behavior (Figure 2b), suggesting a spinal cord mechanism of action for WTD [29, 31–33].

### WTD reduced cytokines and chemokines expressions in spinal cord of SNL mice

Neurogenic inflammation plays an important role in the development of NP [2, 12–14], conspiring with the anti-nociceptive action of WTD in formalin-induced nociception (Figure 2b), we hypothesized that an inhibition of neuro-inflammation mechanism may contribute to WTD anti-hyperalgesia action. For this, we examined the expressions of specific factors in dorsal horn of L5 spinal cord after WTD administration. Supporting the formalin behavioral data, we found SNL significantly increased levels of microglia-expressing cytokines of IL-1β, TNF-α, and astrocyte-releasing chemokines of CCL2, CXCL1, and WTD (6.30-12.60 g/kg) significantly decreased IL-1β, CCL2 and CXCL1 levels, but did not produce obvious change on TNF-α expression (Figure 2c-2f). Taken together, these data suggested that the inhibition of spinal neuro-inflammation may be associated with the anti-hyperalgesia effects of WTD.

### WTD reduced spinal astrocytic IL-1R1, TRAF6 expressions and p JNK levels of SNL mice

It has been well demonstrated that IL-1β and TNF-α, by activating their main receptors of IL-1R and TNFR1 in astrocytes, induce the activation of TRAF6/JNK signaling that maintain the persistence of NP [19]. Typically, type 1 interleukin-1 receptor (IL-1R1) has been regarded as essential for astroglial response and its importance for NP therapy has also been well recognized [34, 35]. Considering the potent inhibitory effects of WTD on IL-1β expression, we postulated the effective inhibitory effects of WTD on IL-1R1/TRAF6/ JNK signaling contribute to the main mechanisms of WTD anti-hyperalgesia action.

**Figure 3:**
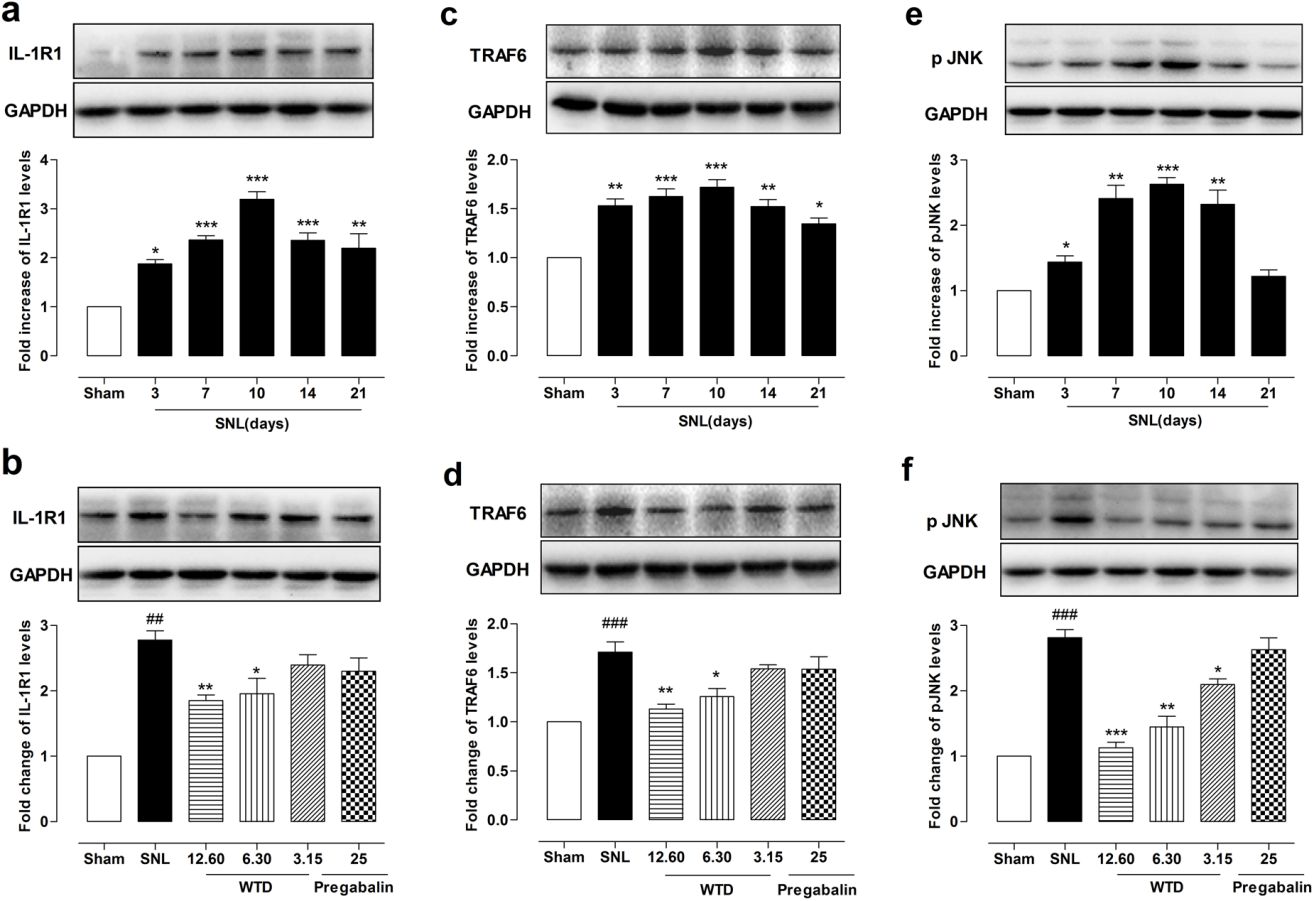
WTD decreased IL-1R1, TRAF6 expressions and p-JNK level in the dorsal horn of L5 spinal cord tissue Time course profile of IL-1R1, TRAF6 expressions, and p-JNK levels in the dorsal horn of the L5 spinal cord in sham- or SNL-operated mice **(a, c, e)**. Chronic administration of WTD (3.15-12.60 g/kg) dose-dependently reduced IL-1R1, TRAF6 expressions and p-JNK levels in spinal cord tissue, while pregabalin (25 mg/kg, p.o.) had no obvious effects on those factors **(b, d, f)**. Data are represented as mean ± SEM. (n=3). ^##^*P*<0.01, ^###^*P*<0.001 *vs*. sham group; ^*^P<0.05, ^**^P<0.01 and ^***^P<0.001 in Figure **3a-3c** *vs*. sham group, in Figure **3b-3f** *vs*. SNL group.

**Figure 4:**
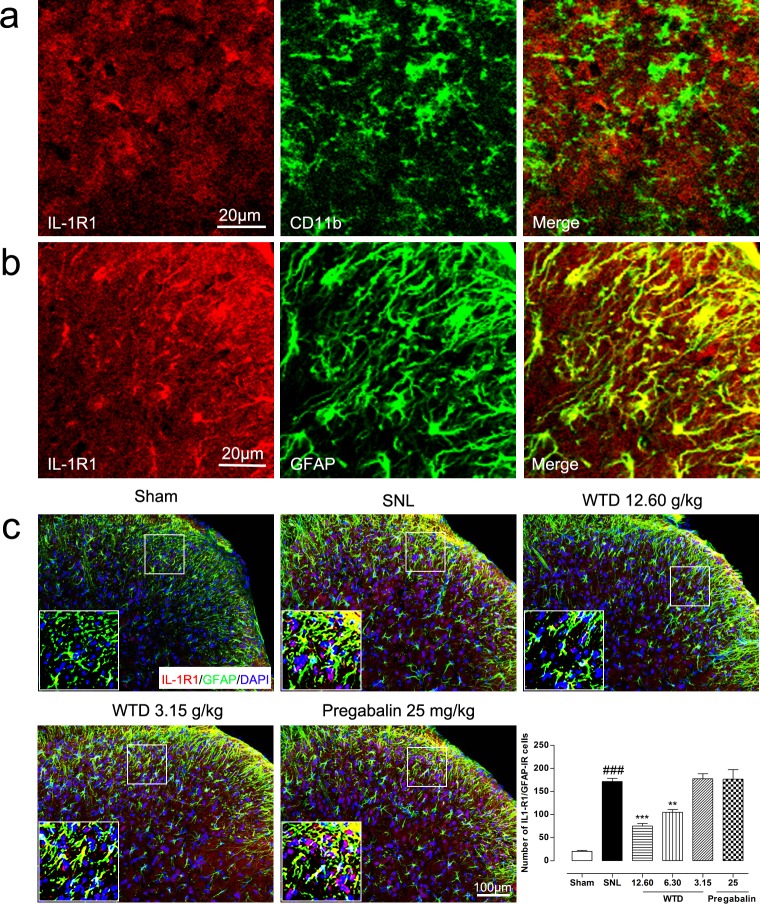
WTD decreased spinal astrocytic expression of IL-1R1 10 days after surgical operation, IL-1R1 has no co-localization with microglia marker of CD11b **(a)**, and were primarily co-localized with astrocyte marker of GFAP **(b)**. Immunofluorescence staining showed WTD (3.15-12.60 g/kg), but not pregabalin (25 mg/kg, p.o.), dose-dependently decreased IL-1R1/GFAP-IR cells in the superficial lamina of L5 spinal cord in SNL mice **(c)**. Data are represented as mean ± SEM. ^###^*P*<0.001 *vs*. sham group, ^**^P<0.01 and ^***^P<0.001 *vs*. SNL group.

For this, we first checked time course profile of IL-1R1 expression in sham- or SNL-operated mice from day 3 to day 21. Western blot analysis demonstrated that SNL significantly increased IL-1R1 expression at day 3, peaking at day 10 and maintaining at least for 21 days, compared to sham-operated group (Figure 3a). Then the peaking point of day 10 was chosen for pharmacodynamics evaluation. Our results demonstrated that WTD (3.15-12.60 g/kg) significantly reduced IL-1R1 expression in a dose-dependent manner, while chronic administration of pregabalin (25 mg/kg, p.o.) did not induce noticeable change on IL-1R1 (Figure 3b). We further checked IL-1R1 expression and distribution by immunofluorescence staining. To examine whether IL-1R1 is expressed in glial cells, we stained IL-1R1 with specific astrocytic marker of glial fibrillary acidic protein (GFAP) or microglial marker CD11b. 10 days after surgical operation, we found IL-1R1-immunoreactivity (IR) had no co-localization with CD11b (Figure 4a), but primarily co-localized with GFAP (Figure 4b). Consistent with the protein expression data, we found IL-1R1 had a very low basal expression in sham-operated animals, SNL significantly increased the number of IL-1R1-IR cells in the superficial lamina of ipsilateral spinal segments, WTD (6.30-12.60 g/kg) also markedly reduced the number of IL-1R1/GFAP-IR astrocytes (Figure 4c), indicating IL-1R1 in astrocytes may involve in the analgesic action of WTD. In accordance with previous reports [36], pregabalin (25 mg/kg) had no obvious inhibitory effect on glial activation or produced noticeable changes on numbers of IL-1R1/GFAP-IR astrocytes (Figure 4c).

**Figure 5:**
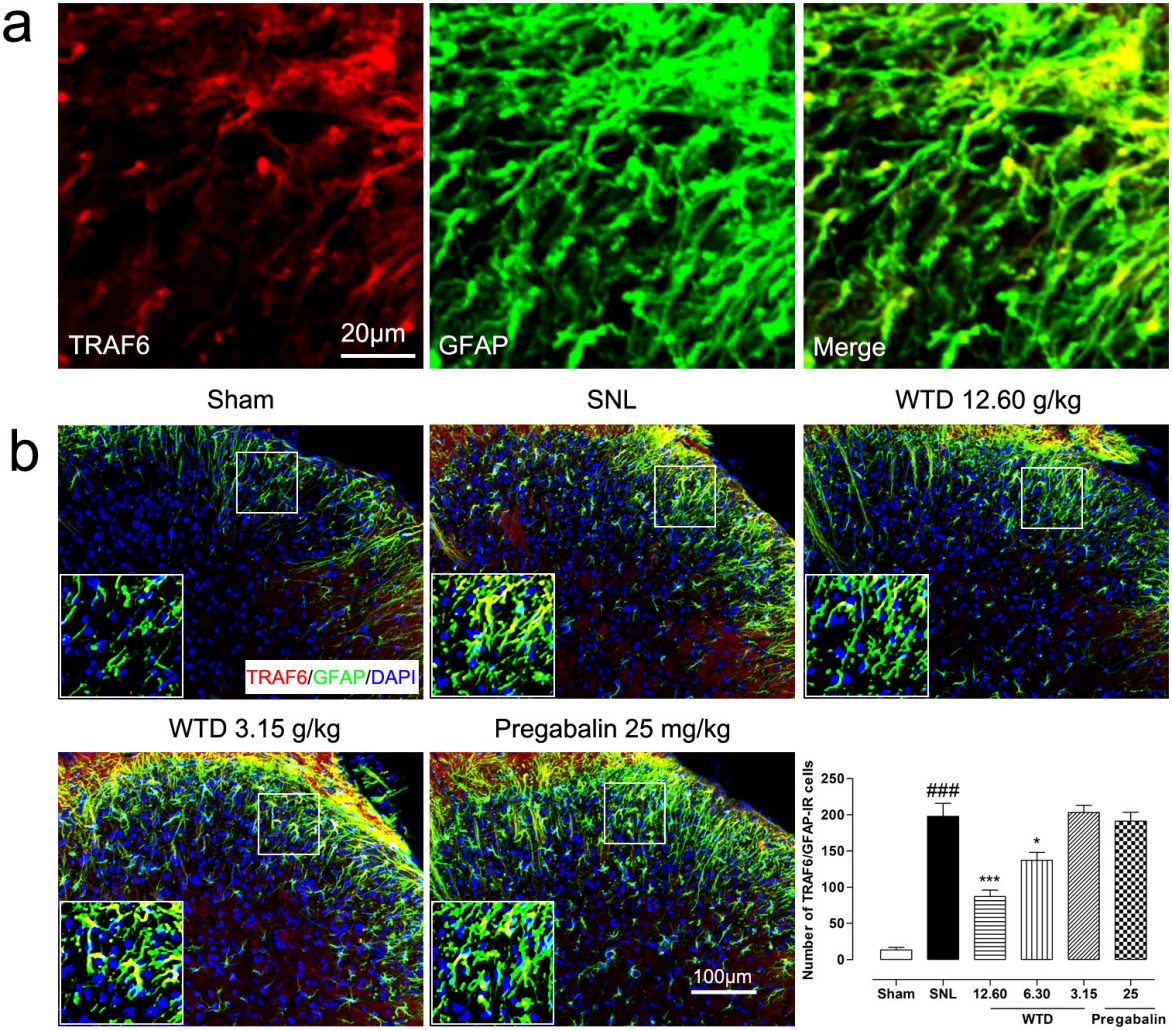
WTD decreased spinal astrocytic expression of TRAF6 10 days after surgical operation, TRAF6 were primarily co-localized with astrocyte **(a)**. Immunofluorescence staining showed WTD (3.15-12.60 g/kg), but not pregabalin (25 mg/kg, p.o.), dose-dependently decreased TRAF6/GFAP-IR cells in the superficial lamina of L5 spinal cord in SNL mice **(b)**. Data are represented as mean ± SEM. ^###^*P*<0.001 *vs*. sham group, ^*^P<0.05 and ^***^P<0.001 *vs*. SNL group.

**Figure 6:**
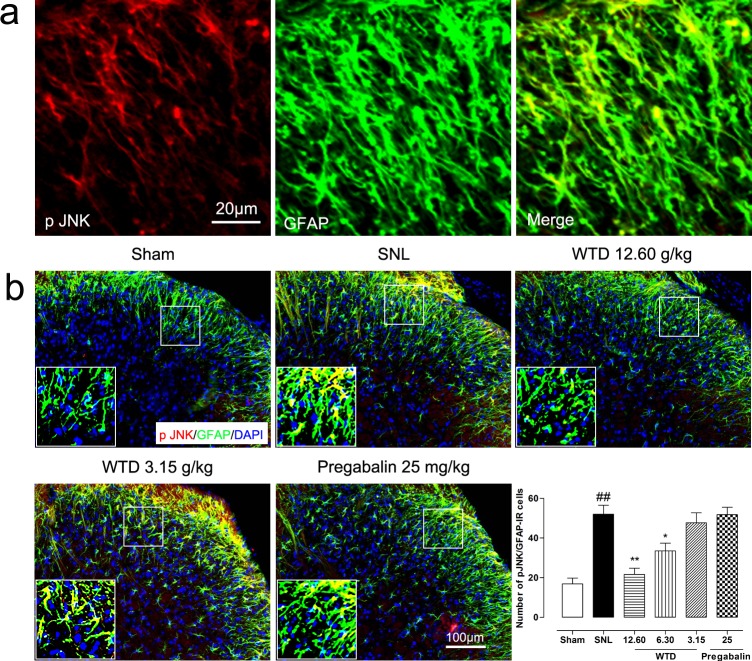
WTD decreased spinal astrocytic levels of p-JNK 10 days after surgical operation, p-JNK were primarily localized in astrocyte **(a)**. Immunofluorescence staining showed WTD (3.15-12.60 g/kg), but not pregabalin (25 mg/kg, p.o.), dose-dependently decreased p-JNK/GFAP-IR cells in the superficial lamina of SNL mice **(b)**. Data are represented as mean ± SEM.^##^*P*<0.01 *vs*. sham group, ^*^P<0.05 and ^**^P<0.01 *vs*. SNL group.

To determine the possible role of the TRAF6 and JNK in the analgesic action of WTD, we further examined the time course profiles of TRAF6 expression and phosphorylation JNK (p-JNK) level of mice from day 3 to day 21. Similar to IL-1R1 data, we also found SNL significantly increased TRAF6 expression and p-JNK level at day 3, both peaking at day 10, maintaining at least for 21 days for TRAF6 and 14 days for p-JNK, compared to sham-operated group (Figure 3c, 3e). 7 days after drug administration, WTD (3.15-12.60 g/kg) significantly reduced TRAF6 expression and p-JNK level in a dose-related manner (Figure 3d, 3f). Accordingly, double staining experiments demonstrated that both TRAF6 and p-JNK were all primarily expressed in astrocytes 10 days after SNL (Figures 5a, 6a). Immunofluorescence staining analysis showed low basal expressions of TRAF6 and low level of p-JNK in sham-operated mice, and SNL significantly increased TRAF6- and p-JNK-IR in the superficial lamina of spinal cord, WTD significantly reduced numbers of TRAF6/GFAP-IR and p-JNK/GFAP-IR astrocytes in a dose-dependent manner (Figures 5b, 6b), indicating TRAF6 or JNK may also contributed to WTD analgesic action. While chronic treatments of pregabalin (25 mg/kg) had no obvious effects on TRAF6/GFAP-IR or significantly reduced the number of p-JNK/GFAP-IR cells (Figures 5b, 6b).

Collectively, these data strongly implicated that IL-1R1/TRAF6/JNK signaling in astrocytes may be involved in the analgesic action of WTD in SNL-induced NP conditions.

### Roles of IL-1R1, TRAF6, JNK in the analgesic effect of WTD under neuropathic pain conditions

**Figure 7:**
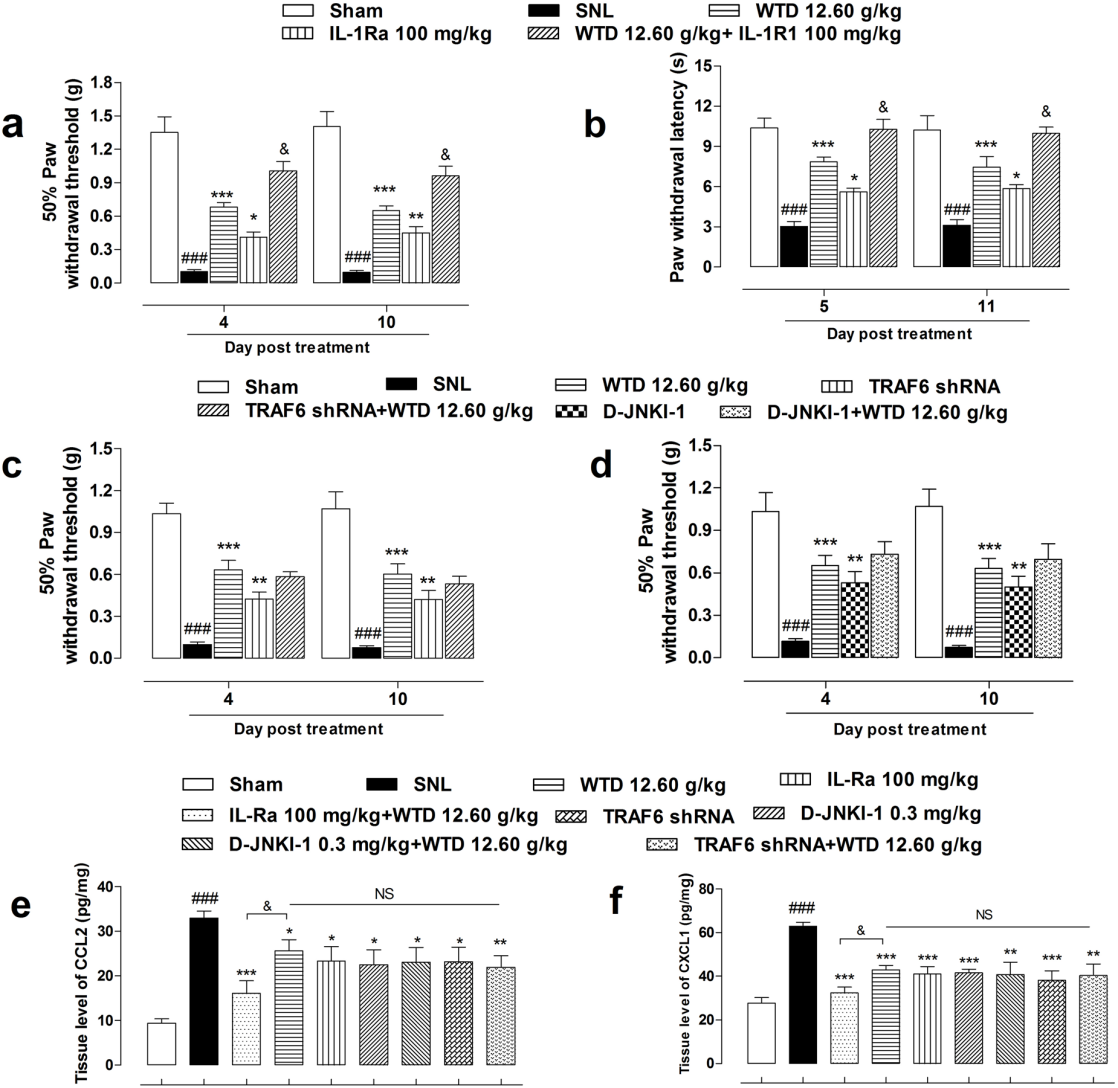
Anti-hyperalgesia and chemokines inhibitory effects of WTD co-administrated with specific inhibitors of IL-1R1/TRAF6/JNK signaling Pretreatment of IL1-Ra (100 mg/kg, i.p., 2 hours before), an inhibitor of IL-1R1, with WTD (12.60 g/kg, p.o., 1 hour before) significantly attenuated SNL induced mechanical allodynia, heat hyperalgesia and increased WTD analgesic effect **(a, b)**. Otherwise, neither co-administration of LV-TRAF6 shRNA (4 μl, intraspinal injection, 3 days prior to test), the specific inhibitor of TRAF6, nor D-JNKI-1 (0.3 mg/kg, i.p., 30 minutes before), the specific inhibitor of JNK with WTD (12.60 g/kg, p.o., 1 hour before) increased WTD's anti-allodynia effect **(c, d)**. Accordingly, co-administration of IL-Ra, but not LV-TRAF6 shRNA or D-JNKI-1 further reduced CCL2 **(e)** or CXCL1 **(f)** expressions. Data are represented as mean ± SEM. (n=6). ^###^*P*<0.001 *vs*. Sham group, ^*^P<0.05, ^**^P<0.01 and ^***^P<0.001 *vs*. SNL group. ^&^*P*<0.01 in **(a-b)** *vs*. WTD (12.60 g/kg) group; in **(e-f)** *vs*. WTD (12.60 g/kg) + IL-Ra (100 mg/kg, i.p.) group, respectively.

To further check the role of IL-1R1/TRAF6/JNK signaling in the anti-hyperalgesia action of WTD, inhibitors of IL-1R1, TRAF6 and JNK were used in behavioral identification tests. As shown in Figure 7a and Supplementary Figure 2a, IL-1Ra, a inhibitor of IL-1R1 (100 mg/kg, i.p.), and WTD (12.60 g/kg, p.o.) significantly reversed mechanical allodynia when drugs were administrated daily for 7 days, interestingly, co-administration of IL-1Ra significantly increased the anti-allodynia effects of WTD, this synergistic effect indicated that the effective inhibition of IL-1R1 was consistent with the analgesic effect of WTD, which further verified in the radiant heat test when pretreatment of IL-1Ra significantly increased PWL in WTD-treated group (Figure 7b and Supplementary Figure 2b). To further assess the roles of TRAF6 and JNK in the analgesic action of WTD, LV-TRAF6 shRNA, a specific inhibitor of TRAF6, and D-JNKI-1, a specific inhibitor of JNK were used, consistent with WTD analgesic effects, both LV-TRAF6 shRNA and D-JNKI-1 showed significant anti-allodynia effects, while no synergistic effect were detected when co-administrated with WTD (Figure 7c, 7d and Supplementary Figure 2c, 2d). Taken all, these behavioral analysis further suggested astrocytic IL-1R1/TRAF6/JNK signaling are involved in the analgesic action of WTD and the effective inhibition of IL-1R1 maybe the key link in this process.

Additionally, co-treatment of IL-1Ra, but not LV-TRAF6 shRNA or D-JNKI-1, with WTD resulted in further inhibition of CCL2 and CXCL1 expressions in the L5 spinal cord of SNL mice (Figure 7e, 7f).

## DISCUSSION

Although WTD significantly reversed CFA-induced hypersensitivity in a TRP-dependent manner [27], little is known about its action on NP. In the present study, by using a SNL-induced NP model, we had the following new findings. First, WTD significantly attenuated mechanical allodynia and heat hyperalgesia in SNL-induced NP without obvious effects on motor functions and body weight loss of animal. Second, WTD decreased formalin (second phase)- but not hot-plate-induced nociception of animals, and significantly reduced spinal IL-1β, CCL2 and CXCL1 expressions. Third, WTD markedly reduced astrocytic expressions of IL-1R1, TRAF6, and p-JNK levels in spinal cord segments. Finally, co-administration of IL-Ra but not LV-TRAF6 shRNA or D-JNKI-1 increased WTD anti-hyperalgesia effect of SNL mice, and further decreased CCL2 and CXCL1 expressions. These data suggest that WTD significantly attenuate NP hyperalgesia without obvious side effect and points to its glia-mediated-neuroinflammation-dependent mechanism possibly via inhibiting spinal astrocytic IL-1R1/TRAF6/JNK signaling. It also suggests that WTD is a potential new drug for the management of NP disorders.

SNL-induced peripheral nerve injury in rodents effectively mimics a clinical chronic NP conditions characterized by mechanical allodynia and thermal hyperalgesia, and has been widely used for NP research and new drug discovery [2, 37]. In this study, we examined the anti-hyperalgesia effects of WTD in SNL-induced NP disorders and found chronic administration of WTD (6.30-12.60 g/kg, p.o.) significantly attenuated SNL-induced mechanical allodynia and heat hyperalgesia without drug tolerance or produced obvious side effects such as impaired locomotor function or body weight loss of mice. To guard against tissue injury, it is imperative to keep normal pain sensation intact for our body to defense potentially damaging stimuli [38]. Importantly, quite different from classic analgesics such as opioids and ion channel blockers, WTD (12.60 g/kg) did not inhibit basal pain perception in sham-operated mice. Thus, our data confirmed the anti-hyperalgesia action of WTD and pointed to its clinical use for NP relive.

Mounting evidence shows that excessive neurogenic inflammation in spinal cord induced by glia activation involved in NP pathological process, and targeting excessive neurogenic inflammation in CNS had been strongly implicated in NP treatment [1–3, 12–14]. Considering the potent anti-inflammatory effects of WTD on peripheral inflammatory conditions [23–27] and its remarkable inhibitory action on neuropathic hyperalgesia, it is reasonable to believe that WTD may also reduce central neuro-inflammation to alleviate NP. To further elucidate the hypothesis, we first undertook a series of initial screening experiments. The hot-plate test is implicated to involve the activation of supraspinal structures, which is a useful tool for screening analgesic drugs that producing central effects [29, 39], and the formalin test is also known to be a useful tool for the initial screening of analgesic drugs which induces two phases of nociceptive behavior, and the second phase is believed to be mediated via central mechanisms characterized by inflammatory mediators modulated within the spinal cord [29, 31–33, 40]. Our data demonstrated that WTD significantly reduced the second phase of formalin-induced nociception, indicating WTD's anti-hyperalgesia action may be spinal cord involved, which was further confirmed by the potent inhibitory effects of WTD on spinal cytokines and chemokines in SNL mice. Additionally, increasing evidence indicates that immune modulators play a powerful role in regulating synaptic plasticity and neuronal excitability that contributes to the persistence of NP [16, 41, 42]. For example, IL-1β, one of the crucial inflammatory cytokines and CCL2 can both enhance excitatory synaptic transmission and reduce inhibitory synaptic transmission and markedly enhances N-methyl-D-aspartic acid (NMDA)-induced currents in dorsal horn neurons [16, 42]. CXCL1 not only elicits pain hypersensitivity but also induces rapid neuronal activation [21], and specific antagonists of these immune modulator attenuated NP hypersensitivity had been demonstrated [2]. Thus, the anti-hyperanalgesia effects of WTD may be mediated, at least in part, by the down-regulation of these immune modulators.

Recently, targeting astrocyte-expressing molecules such as IL-1R [13, 14, 43], TRAF6 [19] or JNK [2, 12–15, 44] has been strongly implicated in persistent pain relive. Previous study demonstrated a regulation of TLR2/TRAF6/Faslg signaling mechanism of WTD in inflammatory conditions [23], combining with the potent inhibitory effects of WTD on gila-releasing factors of IL-1β, CCL2, CXCL1 and the important role of IL-1R1 under NP disorders [34, 35], it is reasonable to postulate that IL-1R1/TRAF6/JNK signaling may be involved in WTD anti-hyperalgesia action. For this, we chose peaking time of day 10 for astrocyte-expressing molecules assessments, and found WTD dose-dependently reduced IL-1R1, TRAF6 expressions, and p JNK levels, particularly, consistent with previous studies, we found IL-1R1, TRAF6 [19] and p-JNK [15] were all primarily localized in spinal astrocytes 10 day after SNL operation, and WTD could also remarkably reduced GFAP/IL-1R1, GFAP/TRAF6-IR and GFAP/p-JNK-IR astrocytes in the superficial lamina of spinal cord, suggesting IL-1R1/TRAF6/JNK signaling in astrocyte correlated to WTD's anti-hyperalgesia action. Further behavioral analysis suggested that co-administrated IL-1Ra, but not LV-TRAF6 shRNA or D-JNKI-1 increased the anti-hyperalgesia effects of WTD, Elisa tests also demonstrated that only IL-Ra further decreased spinal expressions of CCL2 and CXCL1, suggesting IL-1R1 may be the key link contributed to WTD anti-hyperalgesia action, and the effective inhibitions of TRAF6 and p-JNK may be a subsequent mechanism derived from IL-1R1 inhibition.

Throughout the present study, chronic treatment of WTD did not induce drug tolerance in SNL mice, the higher active dose of WTD (12.60 g/kg) did not impair locomotor function, the chronic administration of WTD (3.15-12.60 g/kg) did not manifested obvious body weight loss of mice, and particularly, the higher dose of WTD (12.60 g/kg) did not alter baseline sensory thresholds in sham-operated mice. Therefore, our findings indicated that WTD has a unique role in the normalization of NP. Theoretically, it should be more effective for a drug to target both neurons and glia for pain relief [44]. Considering the TRP-related mechanism of WTD under inflammatory pain conditions [27] and the multi-targets intervention properties of TCM prescriptions [25, 26], it is reasonable to believe that WTD might have better features than other known analgesics for NP treatments.

## MATERIALS AND METHODS

### Animals and surgery

Male ICR mice (8 weeks old) were obtained from Experimental Animal Center of Beijing University, Beijing, China (License No: SCXK-2012-0004). They were kept in a temperature controlled environment (23±1°C), 55±5% relative humidity with a 12h:12h light-dark cycle and with free access to food and water. The experimental procedures were approved by the Research Ethics Committee of China Academy of Chinese Medical Sciences, Beijing, China (Permit Number: 2015-2028) and performed in accordance with the guidelines of the International Association for the Study of Pain (IASP). All efforts were made to demonstrate consistent effects of the drug treatments and minimize the number of animals used and their suffering.

For SNL operations [37], mice were anesthetized with sodium pentobarbital (40-50 mg/kg, i.p.) and a portion of the left L6 transverse process was removed to expose the L4 and L5 spinal nerves, then the L5 spinal nerve was carefully isolated (avoiding injuries to the L4 spinal nerve), and tightly ligated with 6-0 silk thread. Sham-operated mice were subjected to the similar surgical procedures, but no L5 spinal nerve ligation was carried out.

### Experimental design

The experimental designs were as following: (1) 126 mice were randomly divided into 7 groups for verifying the analgesic effects and exploring the related mechanisms of WTD: sham-vehicle group, sham-WTD (12.60 g/kg) group, SNL-vehicle group, SNL-WTD (3.15 g/kg) group, SNL-WTD (6.30 g/kg) group, SNL-WTD (12.60 g/kg) group, and SNL-pregabalin (25 mg/kg) group (n=18 in each group). In each group, 6 mice were used for the tests of mechanical allodynia and heat hyperalgesia, and 9, 4, 5 mice were used for Western blot, experiments of immunofluorescence and enzyme-linked immunosorbent assay (ELISA), respectively. (2) 36 mice were randomly divided into 6 groups to Rota-rod test for assessing whether WTD influenced locomotor function: naïve group, WTD (3.15, 6.30, 12.60 g/kg) groups, pregabalin (25, 50 mg/kg) groups (n=6 in each group). (3) to identify the possible central analgesic effects of WTD, 36 mice were randomly divided into 6 groups and subjected to the hot plate test: naïve group, WTD (3.15, 6.30, 12.60 g/kg) groups, morphine group, and ibuprofen group (n=6 in each group); another 36 mice were subjected to formaldehyde-induced nociception test by the same grouping. (4) to further explore the roles of IL-1R1, TRAF6 and JNK in the analgesic effects of WTD, specific inhibitors of IL-1Ra, LV-TRAF6 shRNA and D-JNKI-1 were administrated, and 36 mice in each test were randomly divided into 5 groups (n=6 in each group): sham group, SNL-vehicle group, SNL-WTD (12.60 g/kg) group, SNL-IL-1Ra or SNL-TRAF6 shRNA or SNL-D-JNKI-1 group, and SNL-WTD (12.60 g/kg)-IL-1Ra or SNL-WTD (12.60 g/kg)-TRAF6 shRNA or SNL-WTD (12.60 g/kg)-D-JNKI-1 group, respectively. In each group, all mice were used for the mechanical allodynia or heat hyperalgesia test and ELISA tests of CCL2 and CXCL1 after drug treatments.

### Drugs and administration

WTD was prepared as we previously described [27, 45]. Briefly, *Radix Aconiti*, *Herba Ephedrae*, *Radix Astragali*, *Raidix Paeoniae Alba* and *Radix Glycytthizae* (with ratio of 6:9:9:9:9) were dried, and homogenized to fine powders together. Then the powdered WTD was immersed in 10 times of distilled water for 1 hour in room temperature, and heated to refluxing for 1.5 hours. After filtration, 8 times of water was added for another 1.5 hours refluxing. The filtered solutions were then concentrated to proper concentration and administered orally (p.o.) in different doses of 3.15, 6.30 and 12.60 g/kg. Morphine was purchased from Northeast Pharmaceutical Group Co., Ltd (Shenyang, China), dissolved in saline, and administered subcutaneously (s.c.). Ibuprofen (140 mg/kg, p.o.), pregabalin (25-50 mg/kg, p.o.) and formalin (20 μl, 1%, i.pl) were of analytical grade obtained from standard commercial suppliers and dissolved in double distilled water or saline. IL-1Ra (anakinra) was purchased from Langen (Germany) and administrated intraperitoneally (i.p.), D-JNKI-1 (i.p.) was purchased from Medchem Express and dissolved in double distilled water.

### Lentiviral vectors production and intraspinal injection

Specific TRAF6 shRNA (5′-TAT GGA TTT GAT GAT GCA G-3′) was designed as previously report [19]. The recombinant lentivirus containing TRAF6 shRNA (LV-TRAF6 shRNA) was packaged using pHS-ASR-ZQ004 vector by Beijing SyngenTech Co., Ltd (Beijing, China). The final titer of LV-TRAF6 shRNA was 8^*^10^8^ TU/ml. The intraspinal injection was performed as described previously [21]. Briefly, on the first day of SNL operation, left L1-L2 vertebral segments was exposed and each animal received 2 injections (2 μl per injection) of LV-TRAF6 shRNA along the L4-L5 dorsal root entry zone (0.8 mm apart and 0.5 mm deep) using a glass micropipette (diameter 50 μm).

### Mechanical allodynia and heat sensitivity analysis

Mice were acclimatized to the testing environments 4 hours every day (8:00-10:00 am and 13:00-15:00 pm) for 3 days. All behavioral tests were performed in a blinded manner. Mechanical allodynia was assessed with von Frey filaments by using Dixon's up-and-down paradigm [46], 30 minutes after habituation in the testing boxes, a series of filaments (0.02-4.0 g, Stoelting) were perpendicularly presented to the central surface of the left hind paw for 2-3 seconds, with force causing slight bent in the hairs. Abrupt withdrawal or flinching behavior of the left hind paw that indicative of positive responses following removal of the filaments were recorded, and PWT was determined.

For heat sensitivity testing, we also habituated mice for 30 minutes in plastic boxes with Hargreaves radiant heat apparatus (Ugo, Basile) and adjusted the baseline of the paw withdrawal latency (PWL) to 9-12 seconds, a cut-off time of 20 seconds was set to prevent tissue injury. Heat sensitivity was assessed by measuring PWLs to the radiant heat stimulus [47].

72 hours after SNL operation, when mechanical allodynia or heat hyperalgesia is fully developed, behavioral analysis were carried out on alternate days from day 4 to day 11, on day 4 and day 5, mechanical allodynia and heat sensitivity were tested 0, 1, 2, 3, 6, 24 hours following WTD (3.15-12.60 g/kg, p.o.), pregabalin (25 mg/kg, p.o.) [48] or vehicle (distilled water, 10 ml/kg, p.o.) treatments, respectively. Time points with optimal drug effects were selected for subsequent behavioral tests, time course profiles of behavioral tests were similar to that of the first day after drugs or vehicle treatments on the ending day.

For behavioral tests of specific inhibitors, 72 hours after SNL, mice were pretreated with vehicle (10ml/kg, p.o, 1 hour prior to test), WTD (12.60 g/kg, p.o., 1 hour prior to test), IL-1Ra (100 mg/kg, i.p., 2 hours prior to test) [43], D-JNKI-1 (0.3 mg/kg, i.p., 30 minutes prior to test)[49] or LV-TRAF6 shRNA (4 μl, 3 days prior to test), and mechanical allodynia or heat hyperanagesia were examined accordingly.

### Rota-rod testing

For locomotor function test, a Rota-rod apparatus (IITC Life Science, Inc.) was applied [50]. Initially, all mice were trained on the Rota-rod for 3 minutes at a speed of 4 rpm 1 day before testing. During the tests, the speed of rotation was programmed accelerated from 4-40 rpm in 5 minutes. The latency of falling from the Rota-rod apparatus was recorded for each animal 0, 1, 3, 6 hours after WTD (3.15-12.60 g/kg, p.o.), pregabalin (25, 50 mg/kg, p.o.) [48] or vehicle treatments.

### Hot plate test and formalin test

Hot plate test was conducted according to previous report [39], with minor modifications. The surface temperature of the hot plate (Ugo, Basile) was set at 50±1°C. Mice were pre-selected 24 hours before testing. The latency of nociceptive response for licking the hind paw or jumping was recorded (in seconds). A cut-off time of 20 seconds was used to avoid paw injury.

For formalin tests, we carried out the procedure as described previously [40], naïve mice were acclimated in the testing Plexiglas chambers for 2 hours every day at least 3 days, and behavioral testing was performed by a blinded observer. On the day of testing, mice were placed in the Plexiglas chambers for 30 minutes and formalin was then administered in the ventral surface of the left hind paw. The time (in seconds) mice spent licking or lifting the affected paw was recorded over the first phase (Phase I, 0-10 minutes) and the second phase (Phase II, 10-45 minutes).

For both tests, mice were pretreated with WTD (3.15-12.60 g/kg, p.o.), ibuprofen (140 mg/kg, p.o., used as a positive control) [51, 52], morphine (2.5 mg/kg, s.c., used as a positive control) [53], or vehicle (10 ml/kg, p.o.) 1 hour before formalin (20 μl, 1%, i.pl) [40] injection.

### ELISA

For Elisa test, mice were transcardially perfused with PBS (4°C, 20 ml) and the left dorsal horns of the L5 spinal cord of mice were rapidly dissected in a “open book” method [54]. Tissue were homogenized in a lysis buffer containing protease and phosphatase inhibitors. The expressions of TNF-α, IL-1β, CCL2 and CXCL1 were measured by using commercially available ELISA kits (BG Products Inc. or Elabscience, China).

### Western blot

Detailed processes of Western blot were carried out as we described before [25–27]. Briefly, after collecting the protein samples [54], 30-50 μg protein was separated on SDS-PAGE gel and transferred to polyvinylidene fluoride membranes. The membranes were then blocked with 5% skim milk powder and incubated overnight at 4°C with gentle shaking against antibodies of IL-1R1 (1:400, anti-rabbit, Abcam), TRAF6 (1:500, anti-rabbit, Abcam), or p-JNK (1:2000, anti-rabbit, Abcam). The loading control membranes were incubated with GAPDH antibody (1:10,000, anti-mouse, Cell Signaling Technology). These membranes were further incubated with corresponding HRP-conjugated secondary antibodies (Zhongshanjinqiao, Beijing, China), developed in ECL (Thermo Scientific) solution in room temperature for 60 seconds, and exposed onto Fusion FX5 system (France, VILBER).

### Immunohistochemistry

7 days after drugs treatments, mice were deeply anesthetized with pentobarbital sodium and rapidly perfused transcardially with 0.9% saline (40 ml, 4°C) followed by 20 ml of 0.16 M PB (4°C, PH=7.4, 10 minutes) containing 4% paraformaldehyde and 1.5% picric acid. After perfusion, the L5 spinal cord segments were removed and post-fixed in the same solution for 4 hours, then cryoprotected in 0.1 M PB containing 15% and 30% sucrose for 24-48 h at 4°C, respectively. Spinal cord sections (20 μm, free-floating) were cut in a cryostat (Leika biosystems) and deposited in cryoprotectant at -80°C.

When testing, the sections were rinsed in PBS (PH=7.2-7.4) 3 times (15 minutes each), blocked with 8% goat serum or BSA in 0.5% Triton-X-100 (life sciences) for 2 hours at room temperature, then the prepared sections were incubated in block solution for 12 hours at 4°C with primary antibodies of IL-1R1 (1:100, anti-rabbit, Abcam), TRAF6 (1:50, anti-rabbit, Santa Cruz), p-JNK (1:200, anti-rabbit, Santa Cruz), GFAP antibody (1:200, anti-mouse, Cell Signaling Technology) and CD11b (1:75, anti-rat, Abcam). After balancing in room temperature for 30 minutes, the sections were then incubated for 1 hour at 37°C with Alexa Fluor® 488 (1:250, Anti-rabbit, Cell Signaling Technology)-, or Alexa Fluor® 594 (Anti-mouse, 1:250, Cell Signaling Technology)-conjugated secondary antibodies. For double immunofluorescence, sections were incubated with a mixture of primary antibodies of GFAP or CD11b with IL-1R1 or TRAF6 or p-JNK as mentioned above, followed by a mixture of Alexa Fluor® 488 or 594-congugated secondary antibodies. After Incubating in DAPI (0.5 μg/ml, Sigma-Aldrich, St. Louis, MO) in PBS for 6 minutes and rinsing with PBS (containing 0.1% tween-20) 3 times (15 minutes each). The stained sections were dried and examined with a FV10-ASW-4.0 software (Olympus, Tokyo, Japan).

### Quantification and statistics

For Western blot quantifications, the density of specific bands of IL-1R1, TRAF6 and p-JNK were measured with a Fusion FX5 imaging analysis system. For GFAP (or CD11b) /IL-1R1, GFAP/TRAF6, GFAP/p-JNK co-localization analysis, 6 non-adjacent sections from the L5-spinal cord segments were randomly selected. Colocalized positive glial cells were counted in the superficial dorsal horn. 4 mice in each group for both analyses. Differences between groups were compared using two-way ANOVA followed by Bonferroni post tests or Student's t-test. The criterion for statistical significance was P<0.05.

## CONCLUSIONS

**Figure 8:**
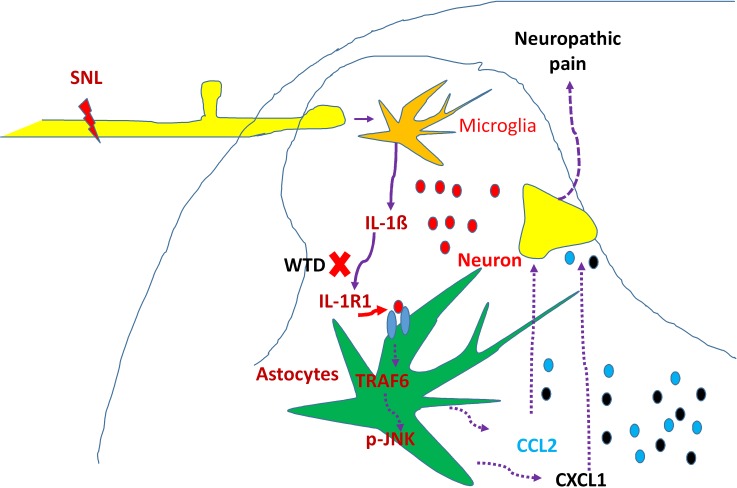
Schematic illustration of possible anti-hyperalgesia mechanisms of WTD under neuropathic pain conditions Peripheral nerve injury induces pro-inflammatory cytokine such as IL-1β, causing the activation of spinal astrocytic IL-1R/TRAF6/JNK signaling and the subsequent releasing of chemokines such as CCL2 and CXCL1, which further facilitate synaptic transmission and enhance NP [19]. The effective inhibition IL-1β and its downstream signaling of IL-1R1/TRAF6/JNK, especially IL-1R1 in astrocytes may be involved in the anti-hyperalgesia effect of WTD under NP disorders.

In summary, our results demonstrated that WTD could significantly attenuate mechanical allodynia and heat hyperalgesia without noticeable side effects under NP conditions, and the effective inhibition of astrocytic IL-1R1/TRAF6/JNK signaling in spinal cord may contribute, at least in part, to WTD anti-hyperanalgesia mechanisms (Figure 8). These findings in the present study add new information and provide evidence for understanding the mechanisms underlying the anti-hyperalgesia effects of WTD, and offer new therapeutic opportunities for chronic pain and related neurological disorders.

## SUPPLEMENTARY MATERIALS FIGURES AND TABLES



## Abbreviations

NP: Neuropathic pain
WTD: Wu-tou decoction
CFA: Complete Freund's adjuvant
SNL: Spinal nerve ligation
CNS: Central nervous system
TNFR: Tumor necrosis factor receptor
IL-1R: Interleukin-1 receptor
IL-1R1: Type 1 interleukin-1 receptor
TRAF6: TNF receptor associated factor 6
JNK: c-Jun N-terminal Kinase
p-JNK: Phosphorylation c-Jun N-terminal Kinase
CCL2: C-C motif chemokine ligand 2
CXCL1: C-X-C motif chemokine ligand 1
GFAP: Glial fibrillary acidic protein
TCM: Traditional Chinese Medicine
TRP: Transient receptor potential ion channel
NMDA: N-methyl-D-aspartic acid
ELISA: Enzyme-linked immunosorbent assay
IR: Immunoreactivity
PWL: Paw withdrawal latency
PWT: 50% paw withdrawal threshold.
