# Supplementary material for: Wu-tou decoction attenuates neuropathic pain via suppressing spinal astrocytic IL-1R1/TRAF6/JNK signaling
Source: Oncotarget. 2017 Oct 6;8(54):92864–79. doi: 10.18632/oncotarget.21638 (PMC5696228; doi:10.18632/oncotarget.21638)
Supplement: Supplementary file 1 [file oncotarget-08-92864-s001.pdf]

Wu-tou decoction attenuates neuropathic pain via suppressing spinal astrocytic IL-1R1/TRAF6/JNK signaling

SUPPLEMENTARY MATERIALS

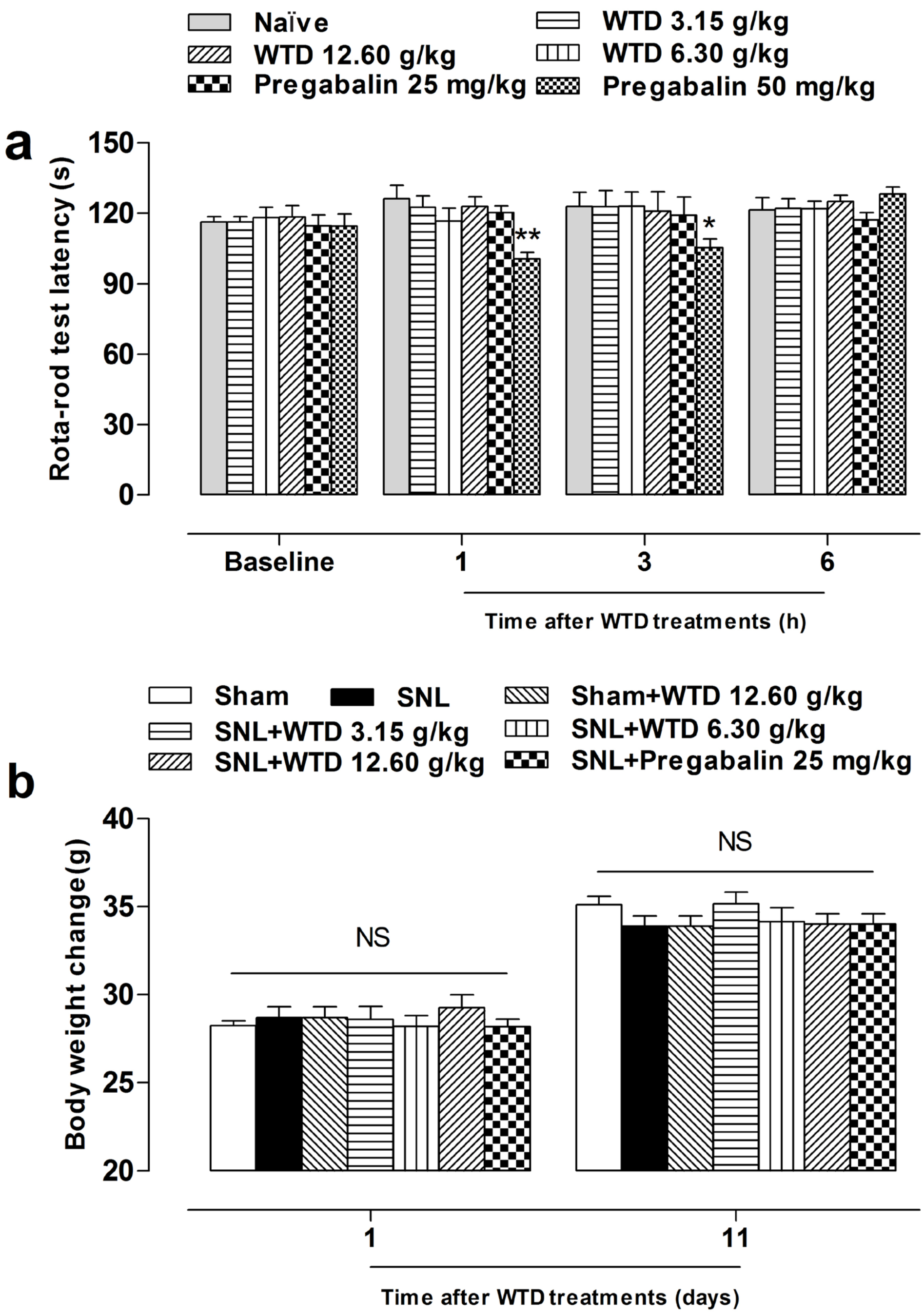

Supplementary Figure 1: Side effects assessments of WTD. WTD (3.15-12.60 g/kg, p.o) and pregabalin (25 mg/kg, p.o.) did not impair the locomotor function of naïve mice in the Rota-rod test 1-6 hours after drug administration, while pregabalin (50 mg/kg, p.o.) significantly reduced the latency time of mice 1and 3 hours post drug treatment **(a)**. WTD did not induce noticeable body weight loss in each group 7 days after drug administration. Data are represented as mean ± SEM. (n=6). $^{*}P<0.05$, $^{**}P<0.01$ in Supplementary Figure 1a *vs.* naïve mice.

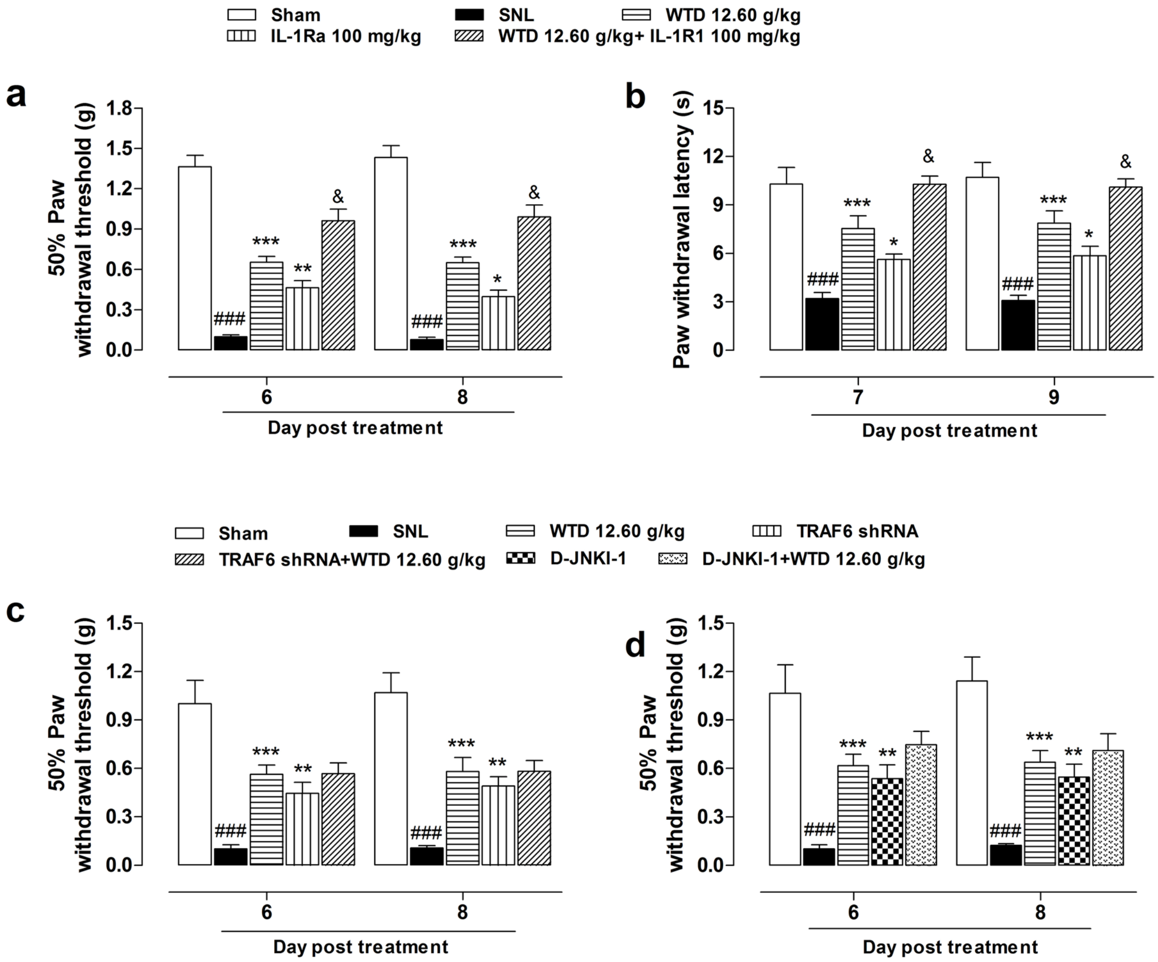

Supplementary Figure 2: Anti-hyperalgesia effects of WTD co-administrated with inhibitors of IL-1R1, TRAF6 or JNK. Pretreatment of IL-Ra (100 mg/kg, i.p., 2 hours before), an specific inhibitor of IL-1R1, with WTD (12.60 g/kg, p.o., 1 hour before) significantly attenuated SNL induced mechanical allodynia, heat hyperalgesia, and increased WTD analgesic effect 5-9 days post drug administration **(a, b)**. Otherwise, neither co-administration of LV-TRAF6-shRNA (4 μl, intraspinal injection, 3 days prior to test), the specific inhibitor of TRAF6, nor D-JNKI-1 (0.3 mg/kg, i.p., 30 minutes before), the specific inhibitor of JNK with WTD (12.60 g/kg, p.o., 1 hour before) increased WTD's anti-allodynia effect on 6th and 8th day post drug administration **(c, d)**. Data are represented as the mean ± SEM. (n=6). $^{###}P<0.001$ *vs.* Sham group, $^{*}P<0.05$, $^{**}P<0.01$ and $^{***}P<0.001$ *vs.* SNL group. $^{\&}P<0.01$ in Supplementary Figure **2a-2b** *vs.* WTD (12.60 g/kg) group.
